# THz guided-mode resonance notch filter with variable filtering strength

**DOI:** 10.1038/s41598-020-80134-2

**Published:** 2021-01-14

**Authors:** Hyeon Sang Bark, Kyu-Ha Jang, Kitae Lee, Young Uk Jeong, Tae-In Jeon

**Affiliations:** 1 Electrical and Electronics Engineering, Korea Maritime and Ocean University, Busan, 49112 Republic of Korea; 2 Interdisciplinary Major of Maritime AI Convergence, Korea Maritime and Ocean University, Busan, 49112 Republic of Korea; 3 Radiation Center for Ultrafast Science, Korea Atomic Energy Research Institute, Daejeon, 34057 Republic of Korea

**Keywords:** Optics and photonics, Physics

## Abstract

In this paper, we propose a terahertz (THz) guided-mode resonance (GMR) notch filter made of a monolithic polyethylene terephthalate (PET) film, which has a monolayer grating structure. The proposed configuration shows both polarization-dependent and polarization-independent notch filter characteristics for the incident THz wave depending on the rotation angle of the second grating film. When the rotation angle is 0°, the filtering strength (transmittance) at resonance frequency changes from 0.4 (0.996) to 99.0% (0.010) according to the incident polarization. The transmittance continuously decreases with increasing rotation angle until 90°. When the rotation angle is 90°, the transmittance converges to 0.065 (± 0.015) independent of the incident wave polarization. When the incident polarization angle ranges from 90° to 180°, paradoxically, the transmittance through the two GMR grating films is greater than the transmittance through only the first GMR grating film due to the enhancement of the vertical component of the THz wave. These results agree well with a calculation using a polar coordinate system.

## Introduction

Guided-mode resonance (GMR) effects in various periodic media are of great scientific and technical interest because of their narrow-band filtering property and simple design. Based on the resonant effect, filters operating in the optical and radio frequency regions have been developed^1–4^. Theoretical and experimental studies have been conducted on various types of dielectric GMR filters to achieve specific functions, such as bandpass filters^5,6^, notch filters^7^, angular-tolerant filters^8^, and polarization-independent grating filters^9–13^. The bandpass, notch, and angular-tolerant filters are polarization-dependent filters that change amplitude, phase, and frequency according to the polarization or angle of the incident wave. These polarization-dependent filters have a one-dimensional (1-D) asymmetrical pattern, such as a strip (or grating). The intensity after passing through the 1-D asymmetrical pattern varies as the square of the cosine of the polarization angle; this is well known as Malus’s law^14^. The polarization-dependent filter is implemented by changing the dimension of the 1-D asymmetrical patterns, such as the period, width, thickness, and filling factor^15^. Polarization-independent filters are also important for many systems and devices, such as sensors^16^, high-power filters^17^, and wave plates^18^. These filters require a two-dimensional (2-D) symmetrically crossed structure based on circular^19,20^, rectangular^21–23^, and rhombic^24^ shapes to demonstrate a constant transmittance for any incident polarization angle. In addition, a cross-stacking of two 2-D GMR gratings are introduced to implement the polarization-independent filter^25^. Since these 2-D structure filters require a substrate for GMR, it is difficult to change the patterns on the substrate. Therefore, polarization-independent filters and polarization-dependent filters cannot be freely compatible with each other, so they must be made and used independently in the optical and radio frequency regions.

Recently, terahertz (THz) frequencies have attracted attention by covering the gap between the optics and radio frequency for science and technology. Polarization-dependent and polarization-independent filters operating at THz frequencies have been developed individually by scaling up and down from the radio frequency and optical frequency ranges, respectively. For example, thin metallic frequency-selective surface (FSS) and metasurface filters have been demonstrated as bandpass filters^26^, notch filters^27^, and angular-tolerant filters^28^. These filters have a very low quality (Q) factor due to the significant losses of the employed substrate. More recently, high Q value GMR filters have been developed for THz frequencies^29,30^. Additionally, the GMR filters were also made to play only one role among polarization-dependent and polarization-independent filters because it is difficult to change the patterns like a filter operating in the optical and radio frequency region. However, in this paper, we report an all-dielectric THz GMR notch filter with variable filtering strength and a high Q value that shows both polarization-dependent and polarization-independent characteristics simply by controlling the angles between the GMR grating films and the incident THz wave polarization. The all-dielectric THz GMR filter is very promising at THz wavelengths because it can be designed and fabricated easily with at a low cost compared to other THz filters using photonic crystal waveguides^31^, parallel-plate waveguides^32,33^, and metamaterials^34^. Moreover, the GMR notch filter made of dielectric materials has a lower refractive index and less absorption loss compared with any other filters, so it can be applied to high-power THz radiation^35,36^ and long-distance THz propagation^37,38^.

## Results

### Filtering strength of a monolayer GMR notch filter

**Figure 1:**
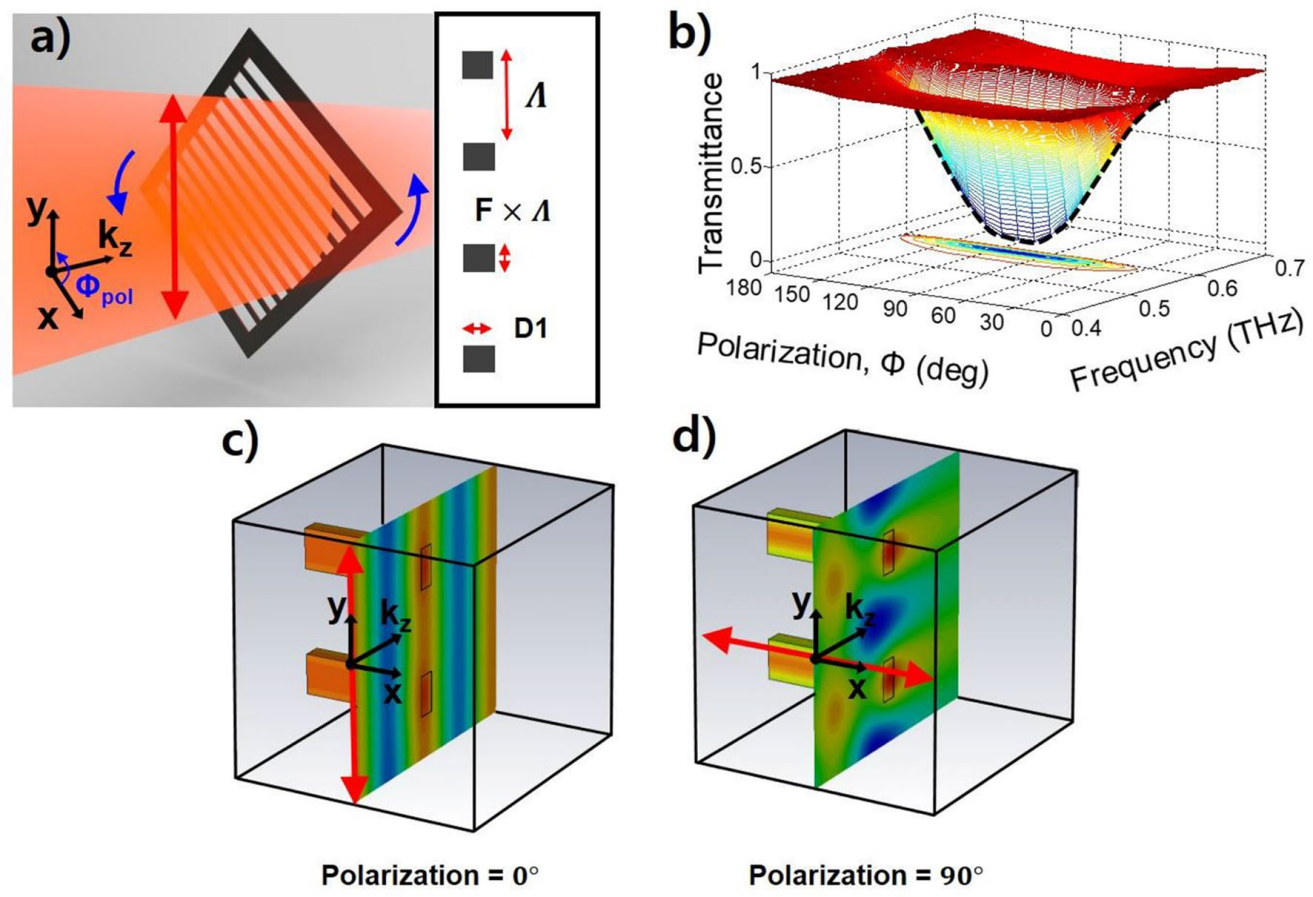
Transmittance properties of a single monolayer GMR notch filter. (**a**) Schematic diagram of the monolayer GMR notch filter at a 45° polarization angle. We set the angle to be 0° when the filter grating and wave polarization are orthogonal. The linearly polarized (y-direction) THz wave enters the monolayer GMR notch filter with a diameter of 25 mm. Instead of turning the polarization of the incident THz wave, the filter rotates counterclockwise. The inset figure shows the cross section of the filter. Each grating pattern consists of a height (D1) of 75 µm, period (Λ) of 510 µm, and filling factor (F) of 32%. (**b**) Measured transmittance according to the polarization angle from 0° to 180° and the wave frequency from 0.4 to 0.7 THz. The dashed black line indicates the numerically calculated transmittance. (**c**) Calculated field distribution of the TM mode when the polarization angle is 0°. The two square-shaped lines in the electric field distribution surface are the outlines of the grating structure. The red arrow indicates the polarization of the incident THz wave. (**d**) Calculated field distribution of the TE mode when the polarization angle is 90°.

The GMR filter is a combination of a grating on the filter surface and slab waveguides of the filter substrate. When a THz wave is incident on the GMR filter, diffraction is produced by the grating. If the angle of the diffracted mode matches the angle of a guided mode produced by the slab waveguide, the coupled THz wave propagates into the slab and creates a strong resonance mode in the spectrum^29^. Recently, monolayer grating films without substrates for slab waveguides have also been found to work as GMR filters in transverse electric (TE) mode^16^ when a THz wave polarized in the same direction as the grating is incident on the GMR filter. We measured the transmittance of the monolayer grating film according to the polarization of the incident THz wave. THz time-domain spectroscopy (THz-TDS) was used to characterize the performance of the GMR filter. In our photoconductive THz-TDS system, the GMR grating film (notch filter) was located between two parabolic mirrors. Figure 1a shows a schematic diagram of the THz wave through a single monolayer GMR notch filter. We designed and fabricated a monolayer GMR notch filter with a polyethylene terephthalate (PET) film with a 75 µm thickness (D1). PET has a refractive index of 1.75 in the THz region^16^. The grating of the GMR filter consisted of a 510 µm period (Λ), a 32% filling factor (F), and 58 grids. The filter size was 3 × 3 cm and was made using femtosecond laser machining (prepared by L2k Co.).

To measure the transmittance according to the polarization of the incident THz wave, the filter was rotated instead of turning the polarization of the incident THz wave. When the vertically polarized THz waves (y-direction) are incident perpendicular to the grating direction of the filter, the polarization angle (Φ) is 0°. The transmittances were measured according to the polarization angle from 0° to 180°, and the resonance frequencies according to the polarization angle appeared at 0.5495 THz (549.5 GHz), as shown in Fig. 1b. When the polarization angle is 0°, which is the transverse magnetic (TM) mode, the maximum transmittance is 0.934. However, in the case of the TE mode whose polarization direction is parallel to grating (Φ = 90°), the THz wave cannot transmit through the filter at the resonance frequency. The minimum transmittance is only 0.070. Therefore, the THz notch filter using the single monolayer GMR grating film can adjust the filtering strength from 7.0% to 93.0%. When the polarized incident beam enters the rotatable GMR filter, the intensity (T) of the measured THz field at the resonance frequency varies as the square of the cosine of the polarization angle. This effect is well known by Malus’s law^14^, which can be expressed as

1 $$ T = T_{0} \cos^{2} (\Phi ), $$

where T_0_ is the intensity of the incident THz field at the resonance frequency. The numerically calculated transmittance according to the polarization angle matches well with the experimental data, as shown by the black solid line of Fig. 1b. Figure 1c,d show the Computer Simulation Technology (CST) simulation results for the electric field distributions around the grating when the polarization angle is 0° (TM mode) and 90° (TE mode), respectively. If the polarization angle is 0°, since the polarization direction of the incident THz wave and the grating direction are perpendicular to each other, then diffraction with respect to the GMR effect does not occur. Therefore, the THz wave freely propagates through the grating, as shown in Fig. 1c. However, if the polarization angle is 90°, then the THz wave is diffracted and guided by the grating, resulting in a GMR effect. Therefore, the THz wave cannot propagate through the grating, as shown in Fig. 1d. The field is guided within the gratings and exhibits a strong resonance, which is a very good notch filter with a high Q value. The transmittance variations according to the polarization angles work as a good polarizer.

### Filtering strength of two-monolayer GMR notch filter

#### Transmittance measurement

**Figure 2:**
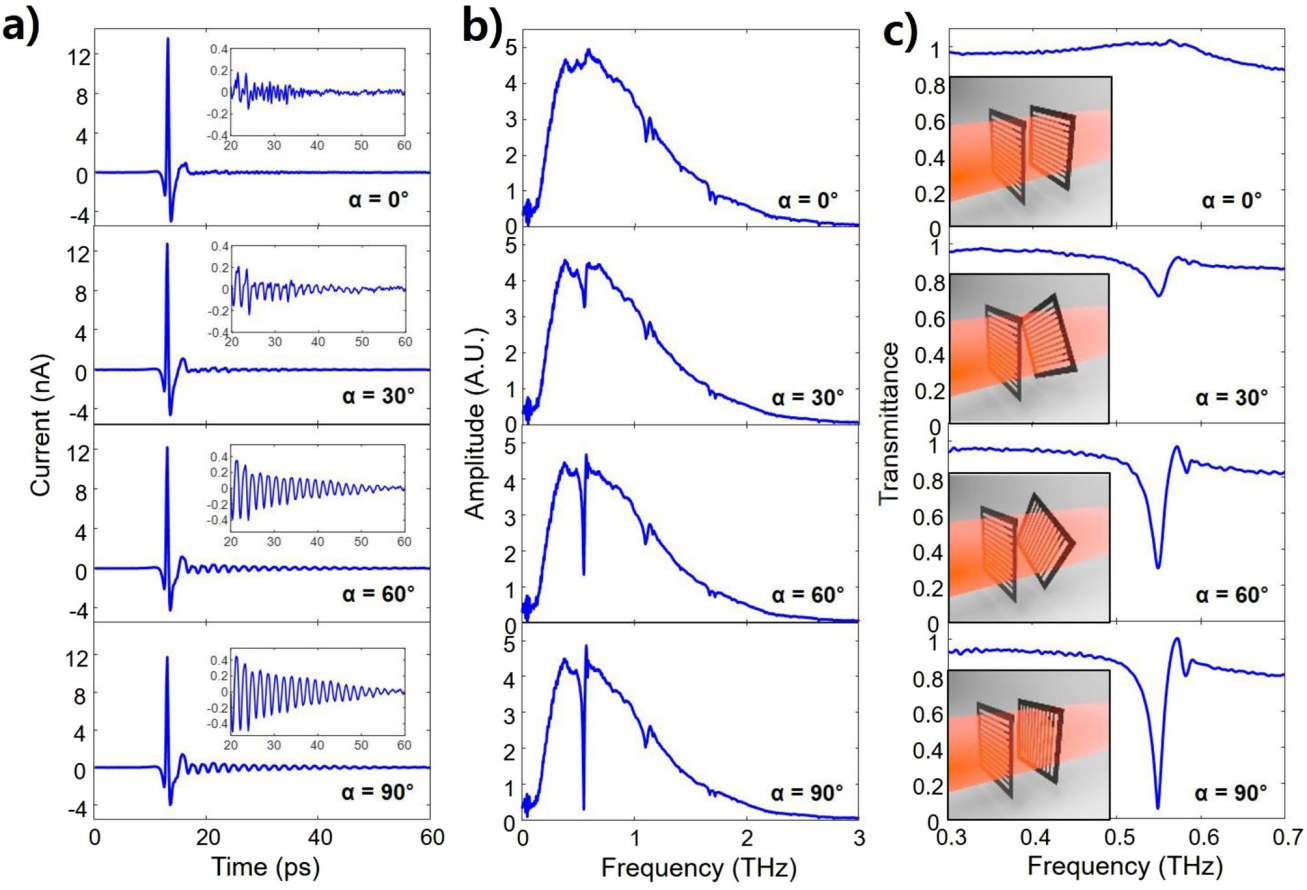
When the angle of the wave polarization and the first grating film is fixed at 0° (Φ = 0), (**a**) Measured THz pulses for different rotation angles of α = 0°, α = 30°, α = 60°, and α = 90°. The inset figures show an expanded window of the oscillations due to the GMR effect after the main THz pulse. (**b**) Corresponding amplitude spectra of the measured THz pulses. (**c**) Corresponding transmittance of the measured amplitude spectra. The inset figures show schematics of the notch filter with two individual monolayer grating films with different rotation.

To have both polarization-dependent and polarization-independent THz notch filter with variable filtering strength, we used two single monolayer GMR grating films, which were placed 8 cm apart to avoid multiple reflections. The first monolayer GMR film was set in the horizontal grid, and the second GMR film was rotated with respect to the grid direction of the first GMR film, which is referred to as the rotation angle (α). We measured different rotation angles from 0° to 90°. When the incident polarization angle (Φ) is fixed at 0°, the measured THz pulses and their spectra with rotation angles of 0°, 30°, 60°, and 90° are shown in Fig. 2. The THz pulses are measured over a scan duration of 160 ps, corresponding to a frequency resolution of 6.25 GHz. A scan consists of 1200 data points with a 20 µm step between data points, corresponding to double-pass (40 µm) time steps of 0.1333 ps between data points. The measured zero-padded THz pulses sum to a total scan length of 1600 ps to increase the spectrum resolution. The inset figures show an expanded time scale (from 20 to 60 ps) of the oscillations due to the GMR effect after the main THz pulse. As the rotation angle increases, the oscillation due to the GMR effect after the main THz pulse increases, as shown in Fig. 2a, resulting in stronger resonance in the spectrum, as shown in Fig. 2b. The resonance frequency is 0.549 THz, which satisfies the diffraction and guiding conditions for GMRs.

Figure 2c shows the transmittance with rotation angles of 0°, 30°, 60°, and 90° when the polarization angle is fixed at 0° (see Section 1 of supplementary information). The inserted figures show a schematic diagram describing the two-monolayer GMR grating films with rotation angles. As the rotation angle increases, the filtering (resonance) depth increases at a resonance frequency of 0.549 THz. When the rotation angle is 0°, the THz wave freely propagates through each GMR film, as shown in Fig. 1c. At this time, the GMR effect does not occur, and the transmittance has a maximum value close to 1, as shown in the first figure (α = 0°) of Fig. 2c. However, by increasing the rotation angle of the second GMR film, the GMR effect increases. The transmittance gradually decreases at the resonance frequency, as shown in the second (α = 30°) and the third (α = 60°) Fig. 2c. Finally, when the rotation angle is 90°, although the THz wave passes freely through the first film, it is blocked by the second film, as shown in Fig. 1d. Therefore, the transmittance at the resonance frequency is a minimum value close to 0 with a high Q value of 37.86, as shown in the fourth (α = 90°) Fig. 2c.

**Figure 3:**
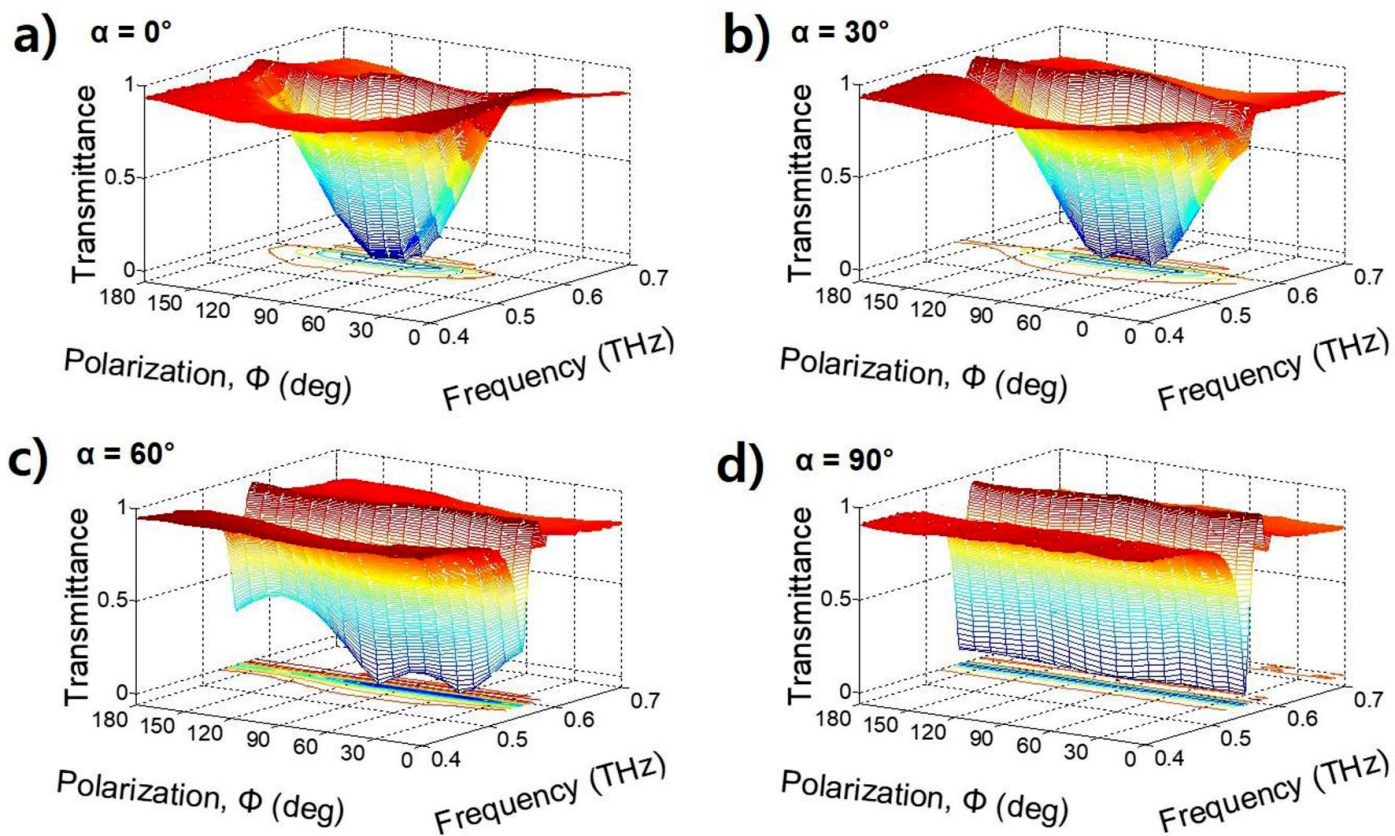
Measured transmittance (filtering strength) according to the polarization angle and THz frequency with different rotation angles of (**a**) α = 0°, (**b**) α = 30°, (**c**) α = 60°, and (**d**) α = 90°. The two GMR films rotate together to measure the transmittance depending on the polarization.

Moreover, instead of rotating the polarization of the incident THz beam, we rotated the two GMR films simultaneously to change the polarization angle from 0° to 180°. According to the change in the polarization angle for rotation angles of 0°, 30°, 60°, and 90°, the transmittances (filtering strengths) are shown in 3-D graphs in Fig. 3a–d, respectively. When the rotation angle is 0° (α = 0°), the transmittance curve at the resonance frequency follows a cosine function, as shown in Fig. 3a. However, the two GMR films have twice the number of grooves compared to a single GMR film, which improves resonance depth^29^. When the polarization angle is 90°, the transmittance of the single GMR film is 0.070; however, the transmittance of the two GMR films is 0.010. Moreover, when the polarization angle is 0°, the transmittance of the two GMR films increases from 0.990 to 0.996. Therefore, the filtering strength (transmittance) for the notch filter with the two-monolayer GMR films at a rotation angle of 0° can be adjusted from 0.4 (0.996) to 99.0% (0.010). When the rotation angle is 30° and 60°, the maximum filtering strength is increased by 22.1% and 59.0%, respectively. Additionally, if the summation of the rotation angle and polarization angle is 90°, then the filtering strength becomes maximum (transmittance becomes minimum). For example, when the rotation angle is 30° and 60° and the polarization angles are 60° and 30°, respectively, the transmittance is minimized, such as 0.008 and 0.006, respectively. As the polarization angle continues to increase, the transmittance increases, and again, the second minimum transmittance appears at a polarization angle of 90°, as shown in Fig. 3b,c. These results form a transmittance bump whose size depends on the rotation angle. When the rotation angle is 90°, the incident THz beam is almost reflected by the two GMR films, as shown in Fig. 3d. In this case, if the polarization angle is 0°, then the incident THz wave freely propagates through the grating of the first GMR filter but cannot propagate through the grating of the second GMR film. In addition, if the polarization angle is 0° < Φ < 180°, then the two GMR films reflect the THz wave in proportion to each polarization angle so that the entire THz wave is blocked at the resonance frequency. The measured transmittance at the 90° rotation angle is 0.065 ± 0.015 at a resonance frequency of 549.0 ± 0.7 GHz, which has a good polarization-independent characteristic. Moreover, the Q value of each resonance is 37.86, which is a high Q value compared to the FSS and metasurface methods^39,40^.

#### Polarization change

**Figure 4:**
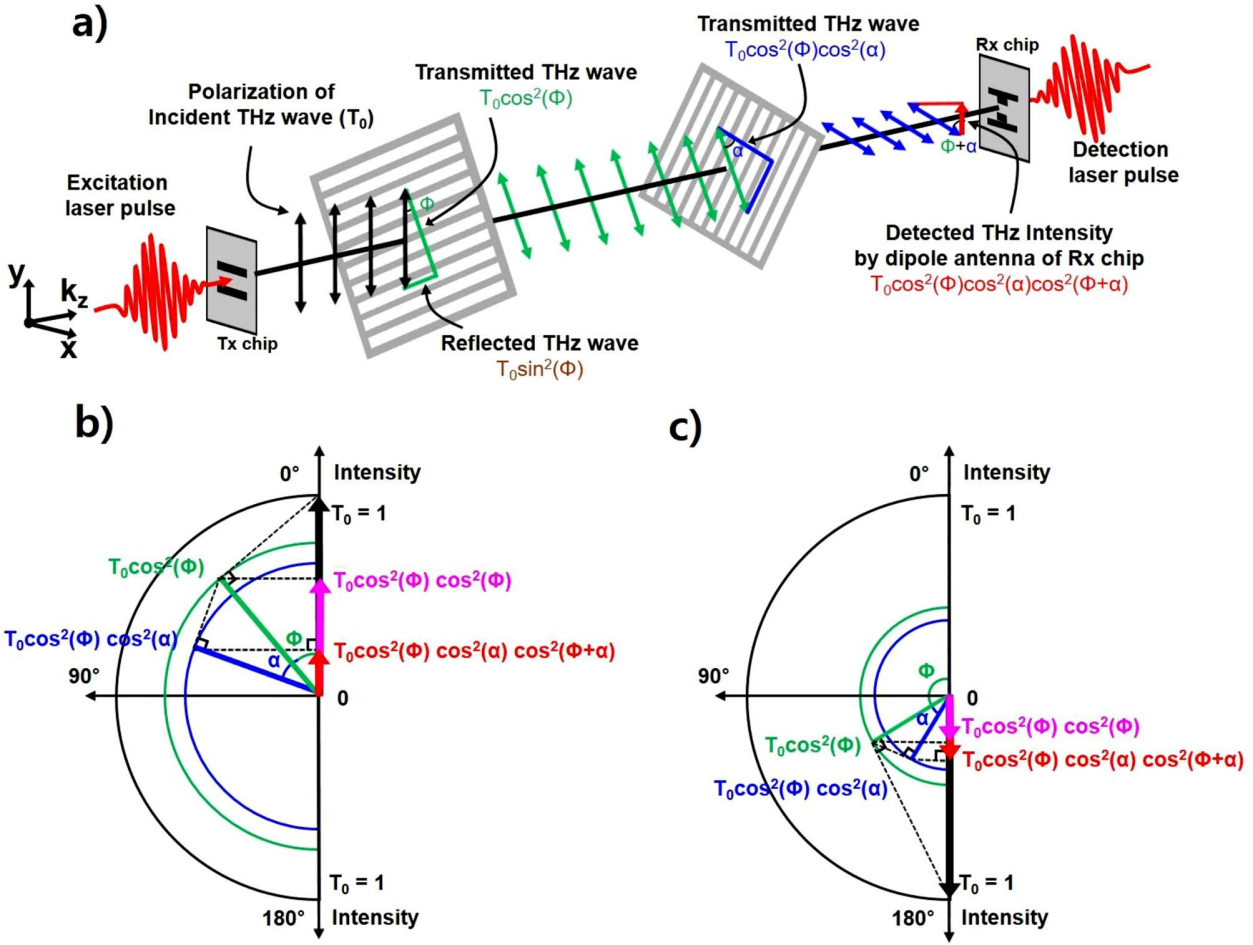
(**a**) Schematic diagram of the polarization change by the two GMR grating films when Φ = 40° and α = 30°. (**b**) The polarization change by the GMR films in a polar coordinate system and the intensity of the THz wave in a Cartesian coordinate system. The green and blue lines indicate the transmitted THz waves after passing through the first and second GMR films, respectively. The arrows indicate the detected THz intensity by the dipole antenna: The polarization range is 0° < Φ < 90°, where Φ = 40° and α = 30°. (**c**) The polarization range is 90° < Φ < 180°, where Φ = 120° and α = 30°.

Figure 4a shows a schematic diagram of the polarization change in the THz waves by two GMR grating films. Since the grating direction of the first GMR film is rotated Φ (for example Φ = 40°) compared to the polarization of the incident THz wave, the incident THz wave passes only the vertical component in the grating direction, and the horizontal component is reflected. Therefore, the transmitted and reflected THz intensities by the first GMR film are T_0_ cos^2^(Φ) and T_0_ sin^2^(Φ), respectively. The transmitted THz beam from the first GMR film enters the second GMR film, which is rotated α (for example α = 30°) compared to the first GMR film. As explained for the first GMR film, the THz wave passes only the vertical component in the grating direction of the second GMR film, and the horizontal component is reflected. Therefore, the transmitted and reflected THz intensities by the first and second GMR films are T_0_ cos^2^(Φ) cos^2^(α) and T_0_ cos^2^(Φ) sin^2^(α), respectively. The dipole direction of the receiver antenna is the same as the polarization of the incident THz beam (y-direction). Finally, the THz wave detected by the receiver antenna is the y-direction component. Therefore, the field detected at the resonance frequency can be expressed as

2 $$ T = T_{0} \left[ {\cos^{2} \left( \Phi \right)\cos^{2} \left( \alpha \right)\cos^{2} \left( {\Phi + \alpha } \right)} \right]^{1/2} + C, $$

where the root is required because the intensity is field square and C is the correction constant. An ideal GMR filter has an infinite number of grids and achieves zero transmittance (maximum resonance depth) at the resonance frequency^29,41^. However, since only approximately 49 grids in the GMR film we used are covered by the incident THz beam, which is 2.5 cm THz beam diameter, the transmittance is not zero. Since the number of grids is limited and the detected field is not zero transmittance, a correction constant is necessary for the compensation of the measured transmittance.

Figure 4b shows the intensity of the THz wave at a polarization angle of 40° and a rotation angle of 30°. The measured intensity using a vertically oriented dipole antenna can be calculated by converting the polar coordinate system to a Cartesian coordinate system, as shown by the vertical arrow lines in the figure. The intensity of the THz wave after passing through the first film is determined by T_0_ cos^2^(Φ). Because the dipole antenna is vertically oriented, the intensity measured by the dipole antenna is T_0_ cos^2^(Φ) cos^2^(Φ), as shown by the pink arrow line in the Cartesian coordinate system. In the same method, the intensity of the THz wave after passing through the second film is T_0_ cos^2^(Φ) cos^2^(α) in the circular coordinate system, and the measured intensity by the dipole antenna is T_0_ cos^2^(Φ) cos^2^(α) cos^2^(Φ + α), as shown by the red arrow line in the Cartesian coordinate system. The intensity of the THz wave passing through two films is less than the intensity of the THz wave passing through only one film. Moreover, when the polarization angle is between 90° and 180°, the intensities of the THz wave passing through the first and second films can be calculated in the same way as shown in Fig. 4c, which shows a polarization angle (Φ) of 120° and a rotation angle (α) of 30°. Paradoxically, the measured intensity of the THz wave after passing through the two GMR films (T_0_ cos^2^(Φ) cos^2^(α) cos^2^(Φ + α) = 0.1406) is larger than that through the first film (T_0_ cos^2^(Φ) cos^2^(Φ) = 0.0625) because the dipole antenna can detect only the vertical component of the THz polarization.

### Comparison of measurement and calculation

**Figure 5:**
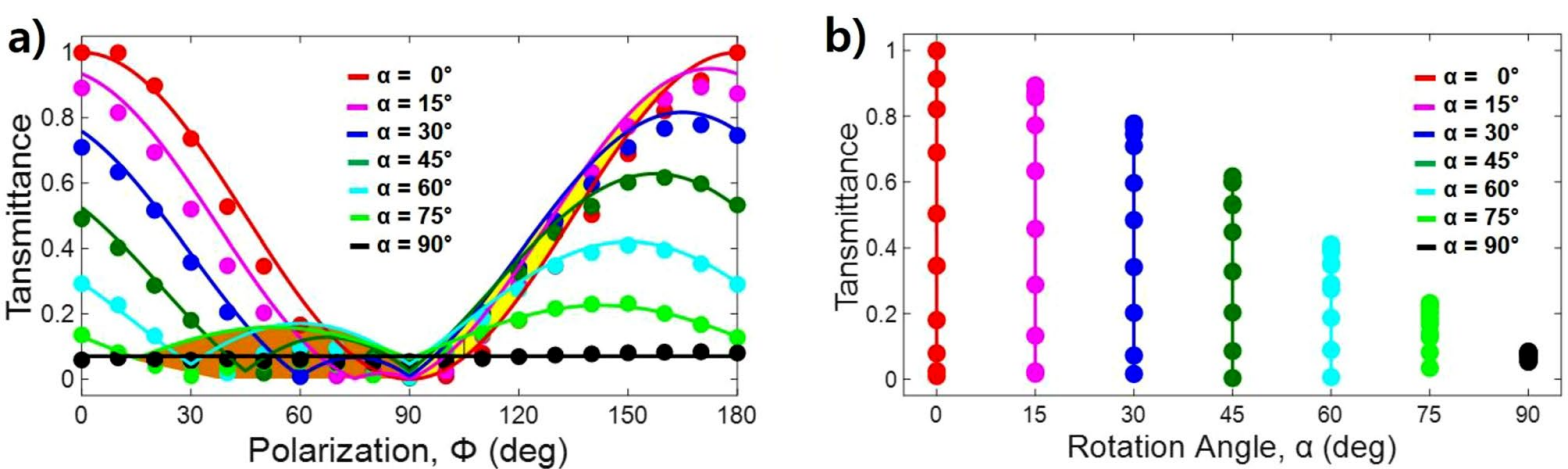
Characterization of the transmittance of two GMR filters. (**a**) Transmittance according to the polarization angle with different rotation angles. Dots and solid lines indicate the measured data and calculated lines by Eq. (2). The measured transmittance data has a maximum deviation of ± 0.02. (**b**) Transmittance variation according to the rotation angle. Dots indicate the measured transmittance according to each 15° rotation angle with 90° < Φ < 180° of the polarization angle. As the dot moves from the lowest to the highest position, the polarization angle increases from 90° to 180°.

The measured and calculated transmittance according to each 15° rotation angle with a 10° polarization angle interval at the resonance frequency is shown in Fig. 5a. The dots and solid lines indicate the measurement and calculated lines by Eq. (2). If the polarization angle is 90°, then the transmittance is always zero because the + y field component is zero after passing through the first GMR film regardless of the rotation angle of the second GMR film. Additionally, when the sum of the polarization angle and the rotation angle is 90°, the transmittance becomes zero because the total angle change by the ideal GMR filter becomes 90° (see Section 2 of supplementary information). If the polarization angle continues to increase and the total angle exceeds 90°, then the -y field components begin to appear. Therefore, the transmittance increases again and then decreases as the polarization angle approaches 90°. That is, transmittance bumps occur in the (90° – α) < Φ < 90° range, as shown in the brown area (collection of bumps) in the figure. Meanwhile, if the polarization angle range is 90° < Φ < 180°, then paradoxically, the -y field component that passed through the two GMR films is larger than that through only the first GMR film, as shown in the yellow area in the figure. For example, when α = 0° and α = 30° for Φ = 120°, the calculated transmittance is 0.250 and 0.375, respectively. Here, if the filters are ideal GMR filters at a zero rotation angle (α = 0°), then the transmittances through one GMR film are equal to the transmittance through two GMR films. Although our GMR grating films have a finite number of grooves, the transmittance through one film is very similar to that through two GMR filters, as shown in Figs. 1b and 3a. Thus, the transmittance through both the first and second GMR films is approximately 150% larger than the transmittance through only the first GMR film. Since the − y field component is enhanced by the rotation angle of the second GMR film, the THz field after passing through the two GMR films appears larger than that through the first GMR film, as shown in the yellow area in the figure. Meanwhile, when the rotation angle is 90°, the angle between the two ideal GMR filters is already 90°. Since the polarization direction of the THz wave passed through the first film and the grating direction of the second film are positioned vertically, the THz waves are totally reflected by the two GMR films. Therefore, the detected transmittance for the ideal GMR filter is always zero regardless of the polarization angle. However, since we used a limited number of gratings in the GMR film, the correction constant should be considered. When the rotation angle is 0°, the gratings of the two films overlap, and the total number of gratings involved in GMR doubles^29^. For this reason, the transmittance is close to zero at a 90° polarization angle. However, when the rotation angle is 90°, the grating involved in the GMR can be obtained as the vector sum of the two gratings, which are perpendicular to each other. Thus, the total number of gratings involved in the GMR is equal to the number of one GMR film. For this reason, the minimum transmittance is slightly higher than zero. The measured transmittance according to the polarization angles at the 90° rotation angle is 0.065 ± 0.015, as shown by the black dots in the figure. Therefore, as the rotation angle increases from 0° to 90°, the correction constant approaches from 0 to 0.065, which is proportional to 0.065 sin^3^(α) in this measurement. The measurement and calculation with the correction constant satisfactorily agree. The correction constant depends on the number of gratings in the GMR filters.

The magnitude of the transmittance increases sequentially with the increase of the polarization angle from 90° to 180°, as shown in Fig. 5b. The dots and solid lines indicate the measurements and fitting lines in Fig. 5a. As the dot moves from the lowest to the highest position, the polarization angle increases from 90° to 180°, respectively. Therefore, the lowest and highest dots indicate polarization angles of 90° and 180°, respectively. This transmittance range shows variable filtering strength according to the rotation angle at the resonance frequency. When the rotation angle is 0°, the filtering strength can be adjusted from 0.4% to 99.0%, which is a very good tunable THz notch filter. When the rotation angle is 90°, the filtering strength (transmittance) is 93.5% (0.065), and the variation range converges within ± 1.5%, which is also a very good polarization-independent characteristic.

**Figure 6:**
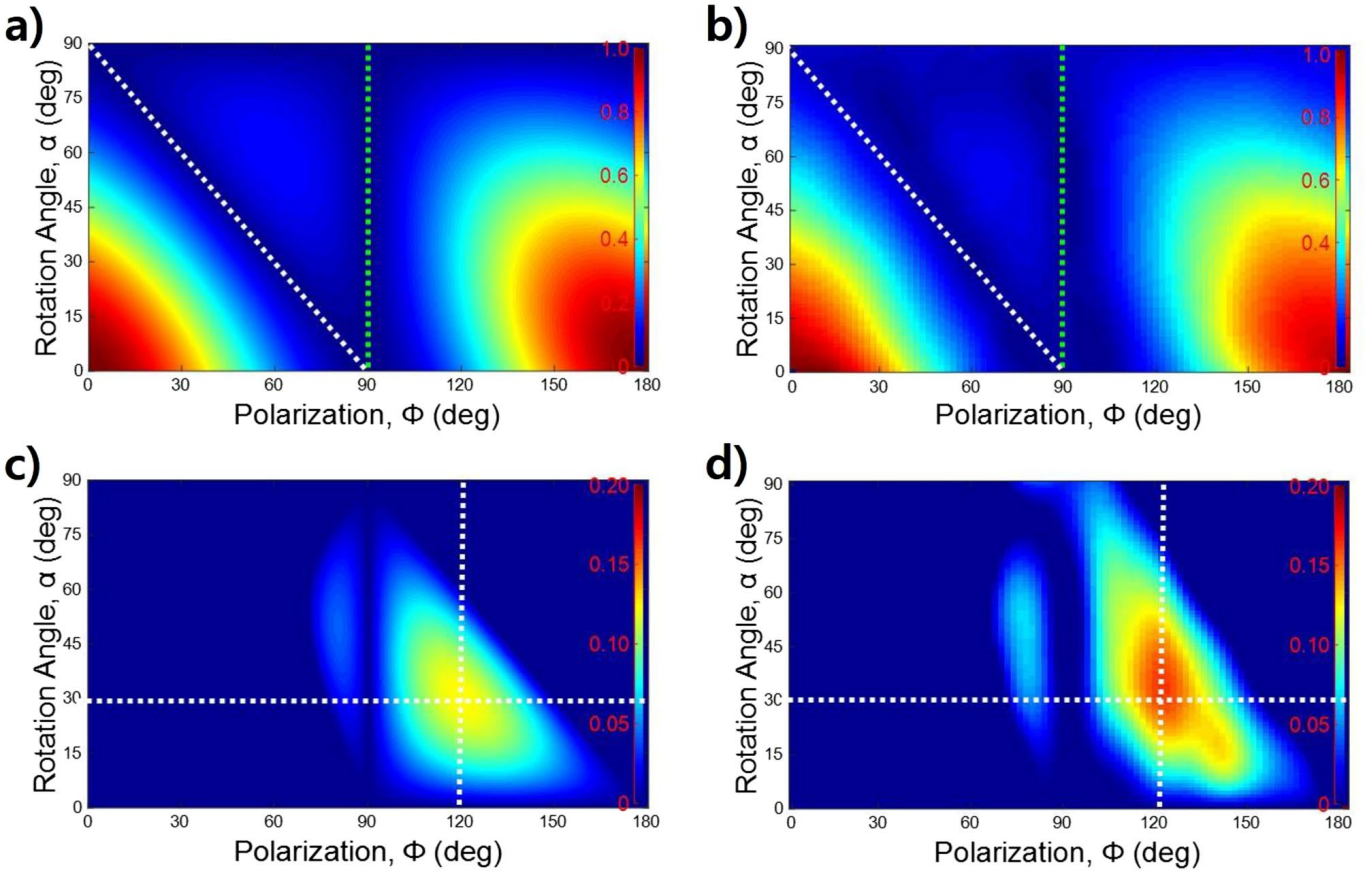
2-D images of transmittance according to polarization angle and rotation angle: (**a**) calculation by Eq. (2); (**b**) measurement. The dashed green and white lines indicate the minimum transmittance when the polarization angle is 90° and when the sum of the polarization angle and the rotation angle is 90°, respectively. 2-D image of paradoxical area as shown in the yellow of Fig. 5a: (**c**) calculation; (**d**) measurement. The intersections of the dashed lines indicate the strongest regions.

2-D images of the calculated and measured transmittance clearly show agreement, as shown in Fig. 6a,b, respectively. The image patterns are very similar. The maximum transmittance exists when the rotation angle is 0° and the polarization angle is 0° and 180°. When the polarization angle is 90°, as shown by the dashed green line in the figures, the transmittance is almost zero regardless of the rotation angle. Additionally, when the sum of the polarization angle and the rotation angle is 90°, as shown by the dashed white line in the figures, the transmittance is also almost zero. Therefore, the filtering strength can be precisely controlled from the maximum to the minimum by adjusting the rotation and polarization angles. The yellow area of Fig. 5a shows that, paradoxically, the transmittance passing through the two GMR films is larger than that passing through only the first GMR film. Figure 6c,d are 2-D images showing paradoxical areas for calculation and measurement, respectively. When the polarization angle is 120° and the rotation angle is 30°, the reversal phenomenon is the strongest, as shown at the intersection of the dashed line. The calculation and measurement results agree well.

## Discussion and conclusions

**Figure 7:**
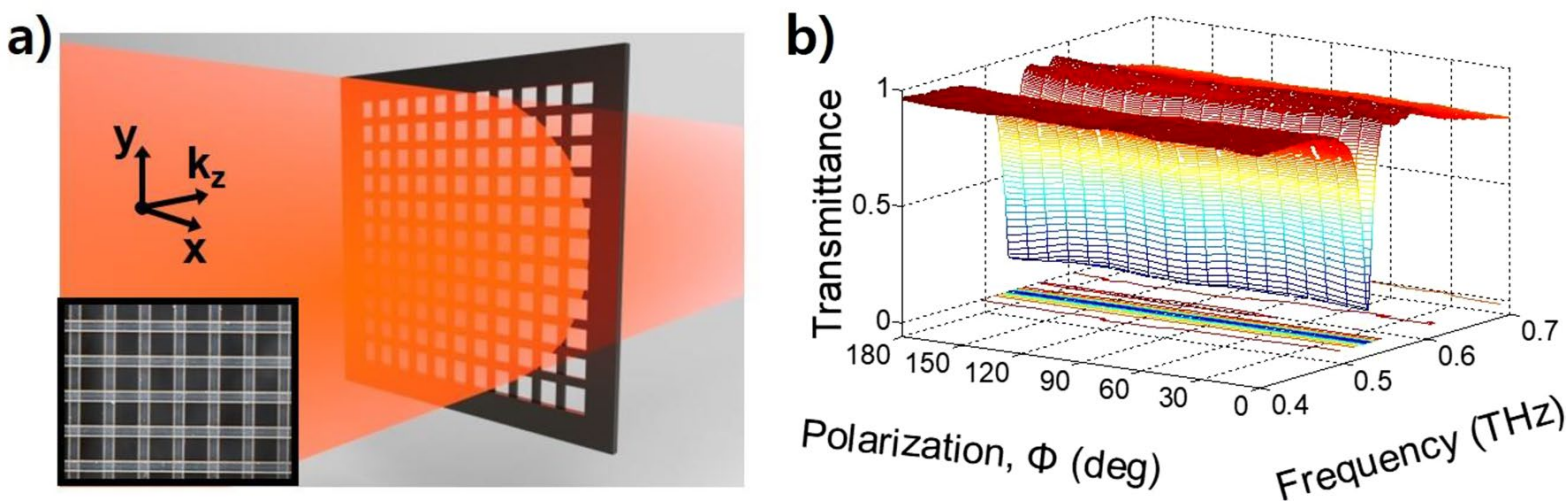
Characterization of the transmittance of a bilayer GMR filter. (**a**) Schematic diagram of the bilayer GMR filter with two-monolayer films attached at an angle of 90°. The inset photo shows the bilayer GMR filter whose gratings form cross strips. (**b**) Measured transmittance according to the polarization angle and THz frequency.

The multiple reflections due to the Fabry–Perot effect were not included in this measurement by providing sufficient spacing between the two GMR filters. Additionally, multiple reflections can be avoided by attaching two GMR films (bilayer GMR filter) (see Section 3 of supplementary information). Figure 7a shows the schematic diagram and photo (inserted figure) of the attached two single monolayer GMR films with a rotation angle of 90°. Due to the periodic gratings in the x and y directions, the transmittance obtained from the bilayer GMR filter is still constant for the incident polarization angles, as shown in Fig. 7b. However, in this case, since the thickness of the bilayer GMR filter is thicker than that of the single monolayer GMR filter, the intensity of the resonance is weakened, and the resonance frequency is shifted at a low frequency^16^. The measured minimum transmittance increases from 0.065 to 0.151, and the resonance frequency shifts from 0.549 to 0.529 THz compared to the two-monolayer GMR notch filter that are separated by 8 cm. Although the minimum transmittance is increased, the bilayer GMR filter still has polarization-independent characteristics.

We proposed a 2-D GMR filter to control the modulation range of the transmittance at the THz resonance frequency. The monolithic grating layer of each 1-D GMR filter is made of a dielectric material, such as a PET film, which is easy to manufacture in various grating forms and has a low cost. When the polarization angle for the ideal 1-D GMR filter changes from 0° to 180°, the transmittance variation changes from 0 to 1 as a function of cos^2^(Φ). However, if the second filter is installed at a rotation angle between 0° and 90° compared to the first filter, then the transmittance ranges for the polarization angles change from 0 to any value less than 1. In particular, when the rotation angle is 90°, the transmittance is always 0 regardless of the polarization angle. In other words, a polarization-independent (insensitive) GMR filter can be performed at a 90° rotation angle. In this study, because a GMR film with a 2.5 cm diameter THz beam and a limited number of grids were used, the polarization-independent transmittance was 0.065 ± 0.015 at 0.5495 THz. When the polarization angle is larger than 90°, paradoxically, the measured THz field after passing through the two GMR films is larger than that through only one GMR film. It is confirmed using a polar coordinate system where the vertical component (y field) formed by the two GMR films is greater than that formed by only the first GMR film. We expect the proposed 2-D GMR filter to be very useful in applications such as THz communications and sensing.

## Methods

### THz-TDS system

We fabricated a conventional THz time-domain spectroscopy (THz-TDS) system using a Ti:sapphire femtosecond laser (Mai Tai, Spectra Physics, USA), providing a 790 nm center wavelength, 60-fs duration at a 80-MHz repetition rate in a beam, with an average power of 12 mW on both the transmitter (Tx) and the receiver (Rx) antennas. The Tx antenna, consisting of coplanar 10 µm wide metal lines with a separation of 80 µm, was fabricated on a semi-insulating gallium arsenide (SI-GaAs) wafer. The laser excitation beam was focused onto the metal–semiconductor interface of the positively biased (80 V) transmission line. The Rx antenna, consisting of two 20 µm wide stubs separated by a 5-*μ*m gap in a coplanar transmission line of two parallel 5 µm wide metal lines with a separation of 10 µm, was fabricated on a low-temperature-grown gallium arsenide (LT-GaAs) wafer. In our photoconductive THz-TDS system, the guided-mode resonance (GMR) filter filters made of a monolithic polyethylene terephthalate film was located in between two parabolic mirrors for transmittance measurement, which can be calculated the spectrum ratio of the output pulse with the GMR filters and a reference pulse without GMR filters.

### Numerical analysis

The numerical simulations are performed by the commercial software, CST Microwave Studio The simulation in Fig. 1c,d, and S6 were performed by the calculations of the CST transient solver, which is based on the finite-integral technique (FIT) method. A hexahedral mesh and periodic boundary conditions (PBC) were applied. The transmission directions were set to total-field and scattered-field. The periodically repeating GMR structures were modeled by infinitely repeating GMR structures, by applying periodic boundaries in both directions.

## Supplementary Information

Supplementary Information.
