# Supplementary material for: THz guided-mode resonance notch filter with variable filtering strength
Source: Sci Rep. 2021 Jan 14;11:1307. doi: 10.1038/s41598-020-80134-2 (PMC7809278; doi:10.1038/s41598-020-80134-2)
Supplement: Supplementary file 1 — Supplementary Information. [file 41598_2020_80134_MOESM1_ESM.pdf]

Supplementary Information

THz Guided-mode resonance notch filter with variable filtering strength

Hyeon Sang Bark[1,3], Kyu-Ha Jang[3], Kitae Lee[3], Young Uk Jeong[3], and Tae-In Jeon[1,2,*]

[1]Electrical and Electronics Engineering, Korea Maritime and Ocean University, Busan 49112, Republic of Korea
[2]Interdisciplinary Major of Maritime AI Convergence, Korea Maritime and Ocean University, Busan 49112, Republic of Korea
[3]adiation Center for Ultrafast Science, Korea Atomic Energy Research Institute, Daejeon 34057, Republic of Korea

* Email (T.-I. Jeon): jeon@kmou.ac.kr

Section 1. Measured transmittance for the rotation angles of two monolayer GMR filters

Figure S1 shows the measured 3-D transmittance according to the polarization angle with different rotation angles. We measured the transmittance at 15° intervals from 0° to 75° and 5° intervals from 70° to 90° to accurately observe the variation in transmittance.

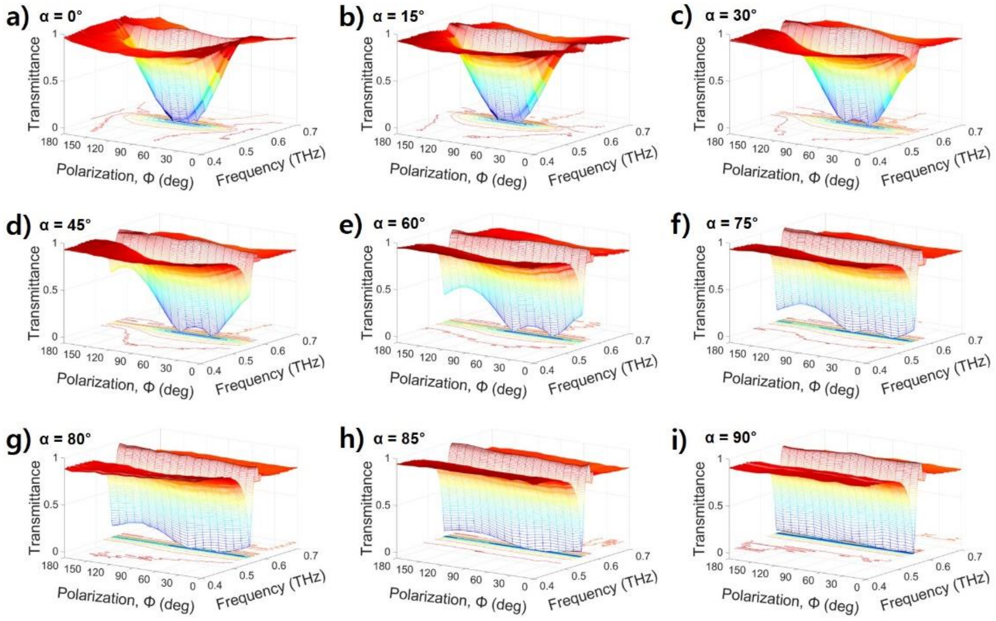

Figure S1. (a-i) Measured 3-D transmittance for two monolayer GMR filters according to the polarization angle with different rotation angles. The two GMR filters are separated by 8 cm.

Section 2. Calculated transmittance according to the dipole antenna direction for ideal GMR filters

When the dipole of the receiver antenna is in the vertical (y) direction and ideal GMR filters are used, the THz field (T_V) detected at the resonance frequency can be expressed as

$$T_V = T_0 \left|\cos(\Phi)\cos(\alpha)\cos(\Phi+\alpha)\right| \quad \text{(S1).}$$

When the dipole of the receiver antenna is in the horizontal (x) direction and ideal GMR filters are used, the THz field (T_H) detected at the resonance frequency can be expressed as

$$T_H = T_0 \left|\cos(\Phi)\cos(\alpha)\sin(\Phi+\alpha)\right| \quad \text{(S2).}$$

If the dipole of the receiver antenna has both vertical and horizontal directions (y and x) and ideal GMR filters are employed, the THz field (T_{V+H}) detected at the resonance frequency can be expressed as

$$T_{V+H} = T_0 \left|\cos(\Phi)\cos(\alpha)\right| \quad \text{(S3).}$$

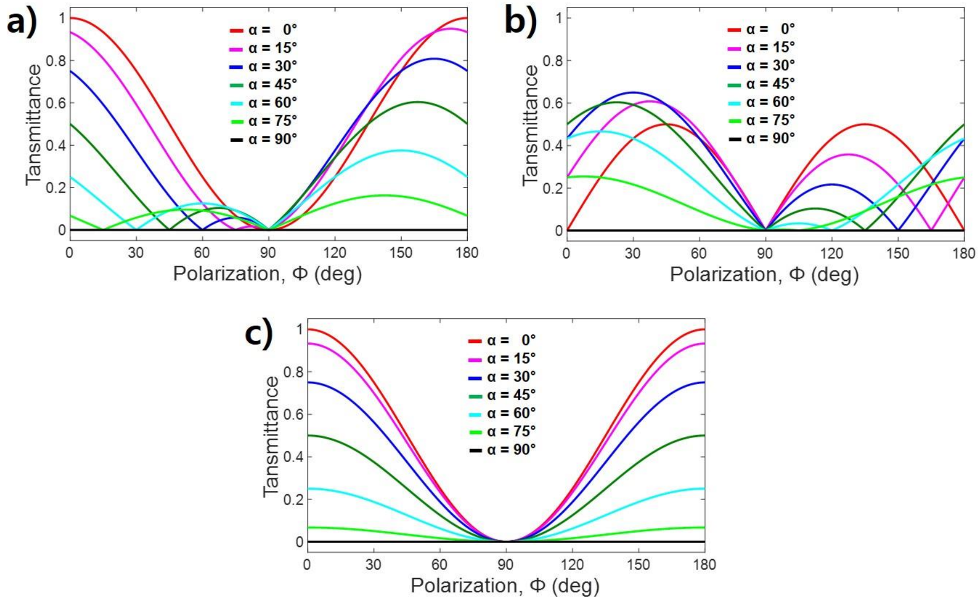

Figure S2. Calculated transmittance according to the dipole antenna direction for two monolayer ideal GMR films. (a) The dipole of the receiver antenna is in the vertical (y) direction. (b) The dipole of the receiver antenna is in the horizontal (x) direction. (c) The dipole of the receiver antenna has both vertical and horizontal directions (y and x).

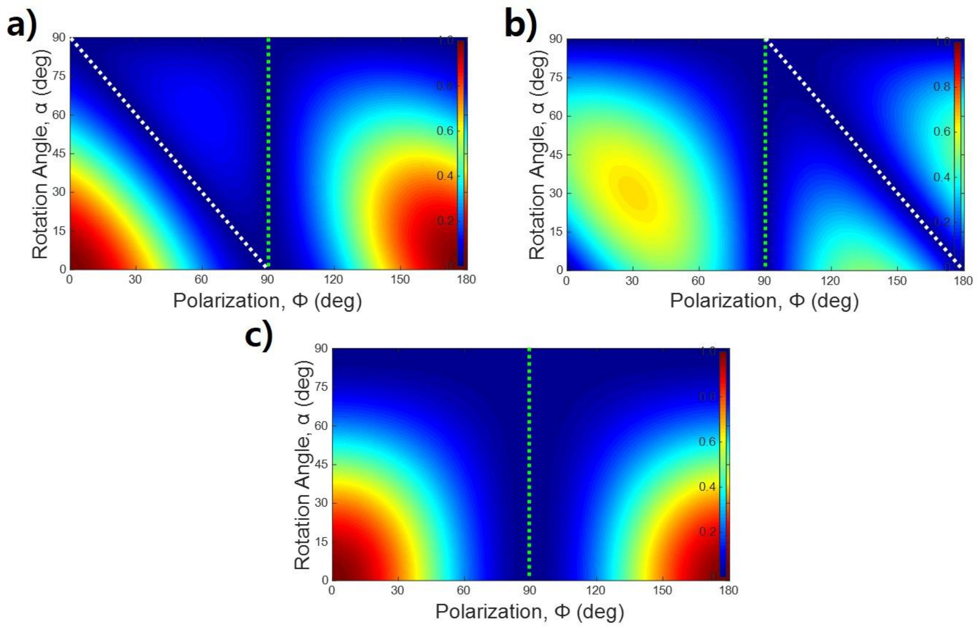

Figure S3. Calculated 2-D transmittance images according to the dipole antenna direction for two monolayer ideal GMR films. The dashed green lines indicate the minimum transmittance at a 90° polarization angle. (a) The dipole of the receiver antenna is in the vertical (y) direction. The dashed white line indicates the minimum transmittance when the sum of the polarization angle and the rotation angle is 90°. (b) The dipole of the receiver antenna is in the horizontal (x) direction. The dashed white line indicates the minimum transmittance when the sum of the polarization angle and the rotation angle is 180°. (c) The dipole of the receiver antenna has both vertical and horizontal (y and x) directions.

Figures S2(a), S2(b), and S2(c) show the calculated transmittance according to the polarization angle with different rotation angles using Eqs. (S1), (S2), and (S3), respectively. Figures S3(a), S3(b), and S3(c) show the calculated 2-D transmittance images using Figs. S2(a), S2(b), and S2(c), respectively. When the polarization angle is 90°, as shown by the dashed green lines in Fig. 3S, the transmittance is zero regardless of the rotation angle. Because the incident THz polarization and the grating direction of the first GMR film are perpendicular, the THz wave is diffracted and guided by the grating, resulting in a GMR effect. Therefore, the THz wave cannot propagate through the grating regardless of the rotation angle of the second GMR film. Additionally, when the sum of the polarization angle and the rotation angle is 90° for the vertically oriented dipole antenna and 180° for the horizontally oriented dipole antenna, the transmittance becomes zero because the total angle change between the two ideal GMR films becomes 90°, as shown by the dashed white lines in Figs. S3(a) and S3(b).

Section 3. Measured transmittance for rotation angles of the bilayer GMR filter

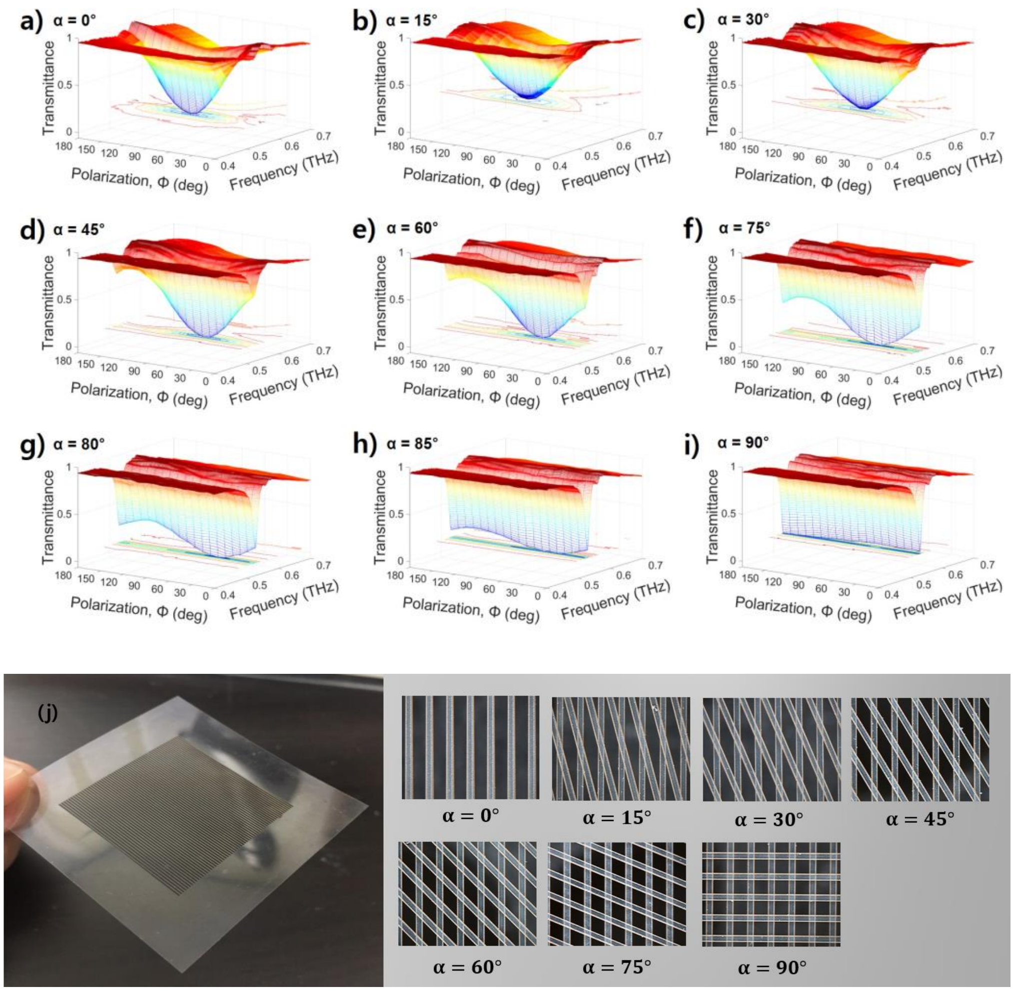

Figure S4. (a-i) Measured 3-D transmittance for a bilayer GMR filter according to the polarization angle. The two GMR films are attached with different rotation angles. (j) Photo of a bilayer GMR film with different rotation angle.

Figure S4 shows the measured 3-D transmittance of a bilayer GMR film according to the polarization angle with different rotation angles. We measured the transmittance at 15° intervals from 0° to 75° and at 5° intervals from 70° to 90° to accurately observe the variation in the transmittance. The transmittance bumps in the $0° < \Phi < 90°$ range shown in Fig. S2(a) are not clearly observed due to the interaction between the overlapping grooves and the thickness of the bilayer GMR film.

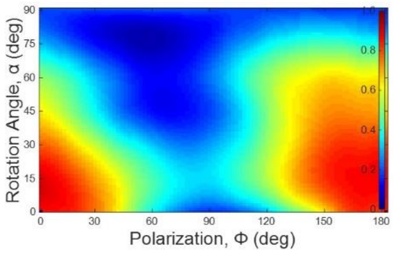

Figure S5. 2-D image of the measured transmittance for a bilayer GMR film

Figure S5 shows a 2-D image of the measured transmittance for nine different rotation angles from 0° to 90°, as shown in Figs. S4(a)–S4(i). When the sum of the polarization angle and the rotation angle is 90°, the minimum and maximum transmittance values do not appear as clearly because of the interaction between the overlapping grooves and the thickness of the bilayer GMR film.

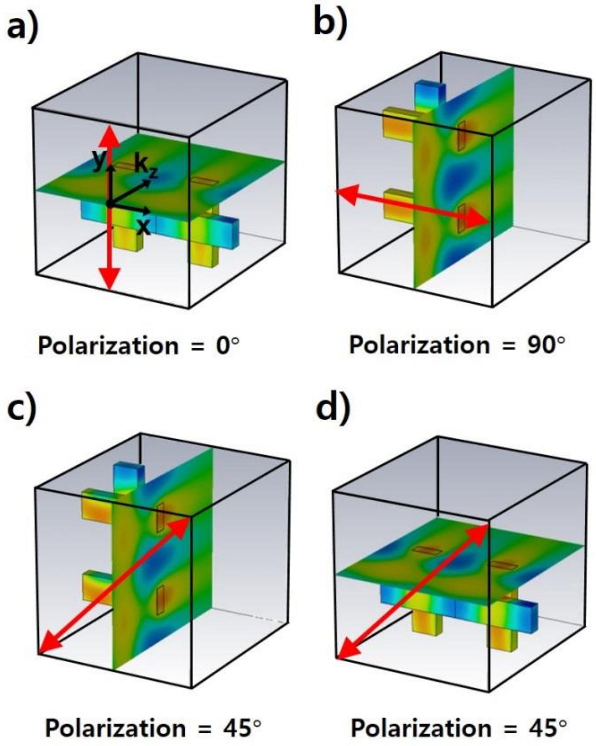

Figure S6. Simulated electric field distribution of a bilayer GMR filter according to the incident THz wave polarization. The arrow indicates the polarization direction. The two square-shaped lines in the electric field distribution surface are the outlines of the grating structure. (a) The polarization angle is 0°, and the electric field is coupled to the second film; (b) the polarization angle is 90°, and the electric field is coupled to the first film; (c) the polarization angle is 45°, and the electric field is coupled to the first film; (d) the polarization angle is 45° and the electric field is coupled to the second film.

We simulated the electric field distribution with CST software to confirm the experimental results at a 0.529 THz frequency when the rotation angle is 90° with polarization angles of 0°,

45°, and 90°, as shown in Fig. 8. When the polarization angle is 0°, the electric field cannot propagate through the second GMR film, as shown in Fig. S6(a). Since the gratings in the first film are coupled with the TM mode, the THz wave is freely transmitted through the first film. However, since the dielectric gratings in the second film are parallel to the THz polarization direction, the gratings are coupled with the TE mode, which prevents THz wave propagation through the second film. Additionally, when the polarization angle is 90°, the opposite phenomenon appears compared with a polarization angle of 0°. The gratings in the first and second films are coupled with the TE and TM modes, respectively. Now, the gratings in the first film prevent THz wave propagation, as shown in Fig. S6(b). Meanwhile, when the polarization angle is 45°, half of the electric field is filtered in the first film, and the other half of the electric field is filtered in the second film, as shown in Fig. S6(c, d). Therefore, the total transmittance reaches 0. In the case of different polarization angles, the transmittance always reaches 0 because the filtering strength is combined by the first and second films. Therefore, this structure can work as a good polarization-independent filter with the maximum filtering strength regardless of the incident THz polarization.
